# Structural basis of mammalian high-mannose *N*-glycan processing by human gut *Bacteroides*

**DOI:** 10.1038/s41467-020-14754-7

**Published:** 2020-02-14

**Authors:** Beatriz Trastoy, Jonathan J. Du, Erik H. Klontz, Chao Li, Javier O. Cifuente, Lai-Xi Wang, Eric J. Sundberg, Marcelo E. Guerin

**Affiliations:** 1 Structural Biology Unit, Center for Cooperative Research in Biosciences (CIC bioGUNE), Basque Research and Technology Alliance (BRTA), Bizkaia Technology Park, Building 801A, 48160 Derio, Spain; 2 Institute of Human Virology, University of Maryland School of Medicine, Baltimore, MD 21201 USA; 3 Department of Microbiology and Immunology, University of Maryland School of Medicine, Baltimore, MD 21201 USA; 4 Program in Molecular Microbiology and Immunology, University of Maryland School of Medicine, Baltimore, MD 21201 USA; 5 Department of Chemistry and Biochemistry, University of Maryland, College Park, MD 20742 USA; 6 Department of Medicine, University of Maryland School of Medicine, Baltimore, MD 21201 USA; 7 IKERBASQUE, Basque Foundation for Science, 48013 Bilbao, Spain; 8 Present Address: Department of Biochemistry, Emory University School of Medicine, Atlanta, GA 30322 USA

**Keywords:** Glycobiology, X-ray crystallography

## Abstract

The human gut microbiota plays a central role not only in regulating the metabolism of nutrients but also promoting immune homeostasis, immune responses and protection against pathogen colonization. The genome of the Gram-negative symbiont *Bacteroides thetaiotaomicron*, a dominant member of the human intestinal microbiota, encodes polysaccharide utilization loci PULs, the apparatus required to orchestrate the degradation of a specific glycan. EndoBT-3987 is a key endo-β-*N*-acetylglucosaminidase (ENGase) that initiates the degradation/processing of mammalian high-mannose-type (HM-type) *N*-glycans in the intestine. Here, we provide structural snapshots of EndoBT-3987, including the unliganded form, the EndoBT-3987-Man_9_GlcNAc_2_Asn substrate complex, and two EndoBT-3987-Man_9_GlcNAc and EndoBT-3987-Man_5_GlcNAc product complexes. In combination with alanine scanning mutagenesis and activity measurements we unveil the molecular mechanism of HM-type recognition and specificity for EndoBT-3987 and an important group of the GH18 ENGases, including EndoH, an enzyme extensively used in biotechnology, and for which the mechanism of substrate recognition was largely unknown.

## Abstract (web summary)

Human gut bacteria depolymerize glycans into their sugar components, which otherwise cannot be processed by their host. Here, the authors characterise the endo-β-*N*-acetylglucosaminidase EndoBT-3987 from the Gram-negative symbiont *Bacteroides thetaiotaomicron* and present the crystal structures of ligand-free EndoBT-3987, a substrate bound complex and product complexes.

## Introduction

The composition and physiology of the gut microbiota plays a pivotal role in human health and disease^1–4^. One major factor that influence the balance of bacterial species in the gut is the influx of glycans into the intestine, mostly from diet as well as host mucosal secretions and secreted epithelial cells^5–8^. The processing of a broad diversity of glycans present in the human gut requires of several glycosidic linkage-specific degradative enzymes^9^. Human intestinal enzymes are capable of fully degrading a small set of glycans containing only one or two different sugar linkages^9^. Gut symbiotic microorganisms provide the complementary enzymatic machinery necessary to depolymerize glycans into their sugar components that otherwise cannot be processed by the host^10,11^. The best studied strategy for glycan acquisition by human gut bacteria is one that is used by members of the phylum *Bacteroidetes*, which represents the main bacterial phylum of the human large bowel. The genomes of *Bacteroidetes* contain polysaccharide utilization loci (PULs)^12^ that encode the apparatus required to utilize multi-subunit carbohydrates, with each PUL orchestrating the degradation of a specific glycan. The gene products of PULs have been termed Sus-like systems because they function by a similar mechanism as does the starch utilization system (Sus) but harbor enzymes that are predicted to target glycans other than starch^13^. The Sus-like systems are widespread among the *Bacteroidetes* members accounting, in many species, for up to one-fifth of their genomes to encode Sus-like pathways, as in *Bacteroides thetaiotaomicron*^14–16^. Sus-like systems are mainly composed of (i) carbohydrate active enzymes, (ii) surface glycan binding proteins, and (iii) a porin that operate the degradation of a certain type of glycan (14). The Sus-like systems identified to date cover the recognition and specificity for all plant and animal tissue glycans that are expected to enter the human gut, as well as the breakdown of *O*-linked and *N*-linked glycans^7,17–25^.

**Fig. 1:**
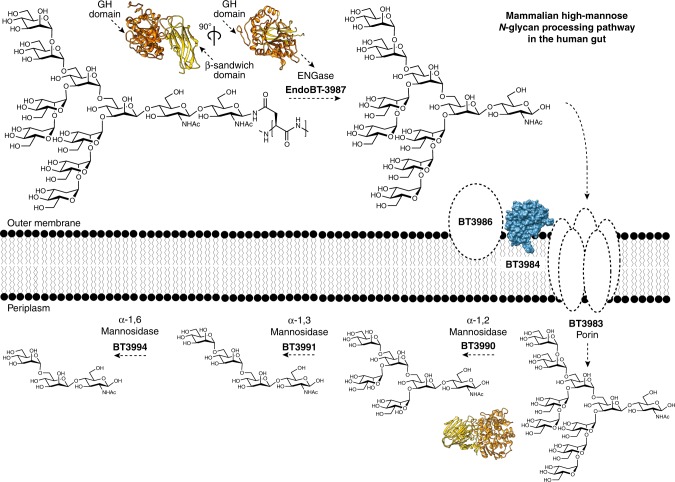
Depolymerization of HM-type *N*-glycans by *B. thetaiotaomicron*. BT3987 deglycosylates HM-glycoproteins in the cell surface. The HM product interacts with the surface glycan binding protein (SGBP), BT3986, and the SusD homolog BT3984 interacts with the reducing end GlcNAc, orientating the glycan into the SusC homolog BT3983 porin. The HM glycan is transported to the periplasm where it is processed by three mannosidases to the trisaccharide Man-α-1,6-Man-β1,4-GlcNAc. Cartoon representation of BT3987 (PDB CODE 6T8I) and BT3990 (PDB CODE 2WVZ), and surface representation of BT3984 (PDB CODE 3CGH; 7).

*B. thetaiotaomicron* encodes a high-mannose mammalian *N*-glycan (HMNG) depolymerizing system, comprised by four enzymes and two surface glycan binding proteins^7^. EndoBT-3987 is a secreted enzyme that initiates the HM degradation pathway and hydrolyzes the oligosaccharide from its polypeptide chain on the cell surface (Fig. 1)^7^. According to the currently accepted model, the released HM is held on the surface of *B. thetaiotaomicron* through the mannose-binding protein BT3986, while BT3984 recognizes the GlcNAc at the reducing end of the glycan and orientates the glycan into the outer membrane porin BT3983) for transport into the periplasm (Fig. 1)^7^. Three periplasmic α-mannosidases hydrolyze the oligosaccharide into the trisaccharide Manα1–6Manβ1–4GlcNAc^7^. BT3990 and BT3991 hydrolyze α1–2Man and α1–3Man linkages, respectively, whereas the terminal undecorated α1–6Man is hydrolyzed by BT3994, which requires GlcNAc at the reducing end for activity^7^. The *B. thetaiotamicron* mutant lacking the extra-cellular σ factor regulator of the HM *N*-glycan PUL reduced the growth of the bacteria showing the importance of *N*-glycan metabolism in these species^7^.

EndoBT-3987 is an endo-β-*N*-acetylglucosaminidase (ENGase) that specifically catalyzes the hydrolysis of the β1–4 linkage between the first two GlcNAc residues of the HM glycans^7,26,27^ but it is not able to hydrolyze complex-type (CT-type) *N*-glycans^27^. ENGases are endoglycosidases that hydrolyze the chitobiose core of *N*-linked glycans (EC 3.2.1.96)^28–30^. This enzyme class comprises glycoside hydrolase family 18 (GH18) and 85 (GH85) of the Carbohydrate-Active Enzymes Database (CAZy; www.cazy.org)^31,32^. GH18 and GH85 family members have been extensively used in past decades to modify the *N*-linked glycan structures in proteins^33^. GH18 ENGases display a wide range of protein specificities, except for EndoS and EndoS2 that are both specific for IgG antibodies^34,35^, but more restricted glycan specificity, and can be divided into three main subfamilies: (i) those that hydrolyze CT-type glycans (e.g., EndoF2, EndoF3, EndoS, EndoS2); (ii) those that hydrolyze HM and hybrid (Hy) *N*-glycans (e.g., EndoH, EndoF1, EndoS2); and (iii) those that hydrolyze HM and CT-type glycans (e.g., EndoS2, EndoBI-1). To our knowledge, EndoS2 is the only example that hydrolyzes the three major groups of *N*-glycans, CT-, HM-, and Hy *N*-glycans on IgG1 antibodies^36^. Perhaps the ENGase most commonly used as enzymatic reagent in glycoprotein research is EndoH from *Streptomyces plicatus*^37^. Since its characterization in 1974^38^, this enzyme has been extensively used as a tool for glycan analysis^39^, to monitor protein trafficking^40–42^ and deglycosylate heterogeneous glycoforms on glycoproteins for crystallographic purposes^43–45^. EndoH has a similar glycan specificity to EndoBT-3987.

The molecular mechanism by which EndoBT-3987 or EndoH specifically recognize HM glycans has yet to be defined, even though several X-ray crystals structures of both enzymes in their unliganded forms have been determined in the past^46,47^. Here, we provide the high-resolution X-ray crystal structures of EndoBT-3987 in its unliganded form, in complex with its substrate, Man_9_GlcNAc_2_Asn, and two of its products, Man_5_GlcNAc and Man_9_GlcNAc. In combination with alanine scanning mutagenesis and hydrolytic activity measurements of EndoBT-3987 point mutants, we present here the comprehensive structural basis of its catalytic and substrate recognition mechanisms, as well as those of other GH18 ENGases that specifically recognize HM-type glycans.

## Results

### Structure of EndoBT-3987 in complex with glycan substrate

**Fig. 2:**
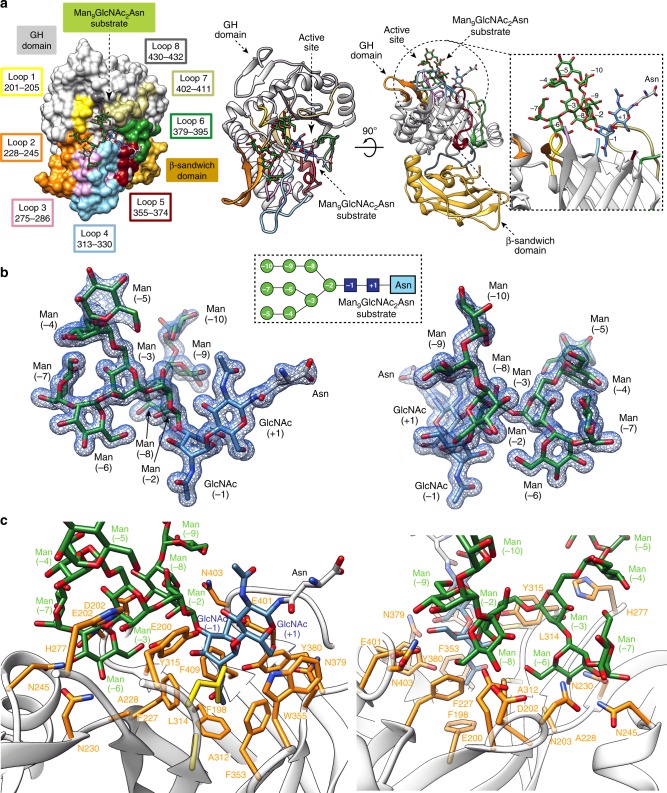
The overall structure of EndoBT-3987 and the substrate Man_9_GlcNAc_2_Asn glycan binding site. **a** Surface representation (left) with annotated domains and GH loops and cartoon representation (center) of two views of the EndoBT-3987_D312A/E314L_-Man_9_GlcNAc_2_Asn crystal structure. On the right panel zoom in of the carbohydrate-binding site of Man_9_GlcNAc_2_Asn. **b** Two views of the electron density of Man_9_GlcNAc_2_Asn substrate shown at 1.0 σ r.m.s deviation. **c** Two views of the key residues of EndoBT-3987 interacting with Man_9_GlcNAc_2_Asn substrate are colored in orange. The mutated catalytic residues D312A and E314A are colored in yellow.

EndoBT-3987 has a predicted signal peptide (residues 1–25; SignalP-5.0) that was removed from the construct. In order to obtain the crystal structure of the enzyme-substrate complex we used a catalytically inactive version of EndoBT-3987, in which the residues Asp312 and Glu314 are mutated to alanine and leucine, respectively (EndoBT-3987_D312A/E314L_; see below for further details). The crystal structure of EndoBT-3987_D312A/E314L_ in complex with the Man_9_GlcNAc_2_Asn substrate was solved by molecular replacement methods (EndoBT-3987_D312A/E314L_-Man_9_GlcNAc_2_Asn complex hereafter; pdb code 6TCV; Fig. 2a; Supplementary Figs. 1 and 2; Supplementary Table 1 and Methods section). This structure represents an example of an enzyme-substrate complex in the GH18 ENGase family. EndoBT-3987_D312A/E314L_-Man_9_GlcNAc_2_Asn crystallized in the *R* 3 space group with one molecule in the asymmetric unit and diffracted to a maximum resolution of 1.3 Å (Supplementary Table 1). Residues 26–41 were not visible in the structure. The full-length EndoBT-3987 comprises two domains from the N- to the C-terminus: (i) a β-sandwich domain (42–179) followed by a short linker (residues 180–193) that continues to (ii) a GH domain (residues 194–476).

The EndoBT-3987 GH domain adopts the conserved (α/β)_8_-barrel topology typical of enzymes from the GH18 family, with a shallow pocket in which one molecule of the Man_9_GlcNAc_2_Asn substrate is unambiguously identified in the crystal structure (Fig. 2b, c). Specifically, the Man_9_GlcNAc_2_Asn substrate is located in the center of the (α/β)_8_-barrel, flanked by the connecting loops β10–α2 (loop 1; residues 201–205), β11–α3 (loop 2; residues 228–245), β14–α4 (loop 3; residues 275–286), β15–α5 (loop 4; residues 313–330), β16–β17 (loop 5; residues 355–374), β17– β18 (loop 6; residues 379–395), β18–α6 (loop 7; residues 402–411), and β19–α7 (loop 8; residues 430–432) (Fig. 2a–c). The Asn residue is completely exposed to the solvent, accounting for the ability of EndoBT-3987 to process HM-type glycans attached to a broad spectrum of proteins^7,26,27^. The O3 and O6 atoms of the first GlcNAc (+1) residue make hydrogen bonds with the side chains of Y315 and N379, respectively. The GlcNAc (+1) residue is stabilized by an additional van der Waals interaction with the side chain of W355. The GlcNAc (−1) residue is in a skew boat conformation (^1^*S*_5_), with the C2-acetamido group pointed toward the mutated catalytic residue D312A. The skew boat conformation of GlcNAc (−1) is stabilized by hydrogen bonds between the O3 atom and the side chain of Y315, the N2 atom of the acetamide group and the side chain of Y380, and the O6 atom and the side chain of E401. The hydrophobic residues F198, F227, F353, and F429 located at the top of the corresponding β10, β11, β16, and β19 β-strands of the barrel, respectively, form the floor of the binding pocket (Fig. 2d). The O_2_ atom of the central Man (−2) residue makes a hydrogen bond with the side chain of E200. The Manα1–2Manα1–6(Manα1–2Manα1–3)Manα1–6 antenna (antenna 1), mainly interacts with loops 1, 2, 3, and 4, whereas the Manα1–2Manα1–2Manα1–3 antenna (antenna 2) interacts with loops 1 and 7 (Fig. 2). The O2 atom of Man (−3), which bisects antenna 1 into two additional sub-antennae, antenna α1,6′ (antenna 1a; Man (−4) and (−5) residues), and antenna α1,3′ (antenna 1b; Man (−6) and (−7) residues; Fig. 2), makes a hydrogen bond with the Nε2 atom of H277. The O4 atom makes a hydrogen bond with O6 of Man (−7). The Man (−3) residue is stabilized by an additional van der Waals interaction with the side chain of Y315. The Man residues of antenna 1a are solvent exposed, Man (−4) and Man (−5) make van der Waals interactions with H277. In contrast, the antenna 1b is buried in the substrate binding pocket (Fig. 2). The O2 atom of the Man (−6) residue of antenna 1b makes electrostatic interactions with the side chains of N202 and N245; whereas the O3 makes a hydrogen bond with the side chain of N230, located in β12 of the β12–β13 hairpin, and also interacts with the side chain N245. The O4 atom makes a hydrogen bond with the main chain of A228 and also interacts with the side chain of N230. Finally, the O6 atom makes a hydrogen bond with the side chain of E200. The Man (−7) residue of the antenna 1b is mainly solvent exposed. The O4 and O6 atoms of the Man (−8) residue located in antenna 2, make hydrogen bond with the side chains of D203 and E200, respectively. In addition, the O4 of Man (−9) makes electrostatic interaction with the side chain of N403, while Man (−10) is completely solvent exposed. For more information about the catalytic cycle of EndoBT-3987, please see Supplementary Note 1 and Supplementary Fig. 3.

### Structure of EndoBT-3987 in complex with two glycan products

**Fig. 3:**
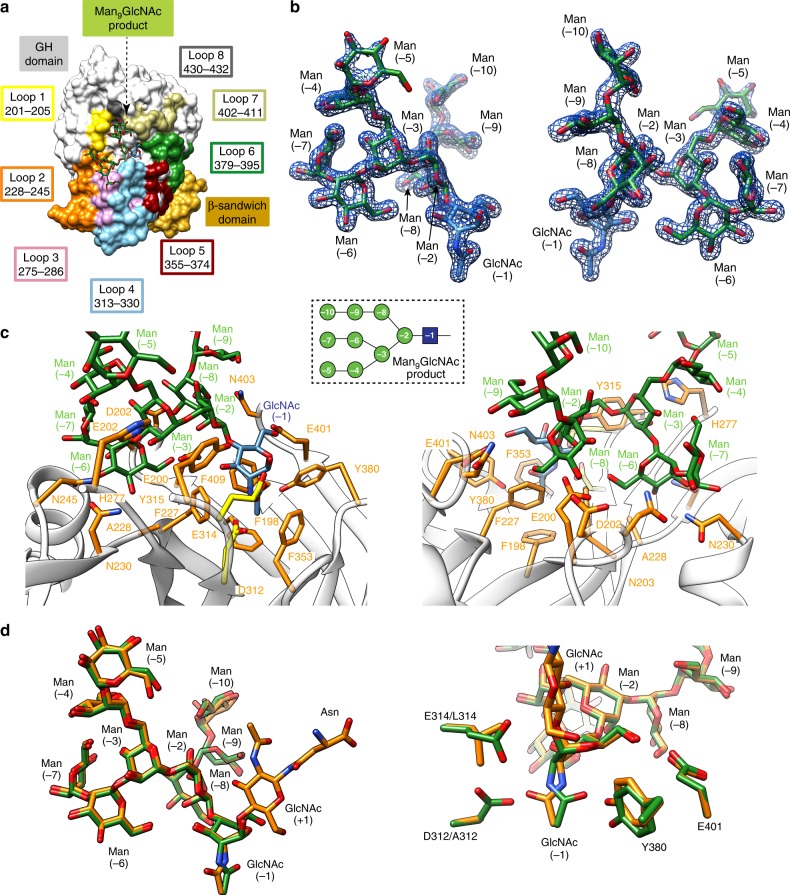
The product Man_9_ glycan binding site. **a** Surface representation with annotated domains and GH loops of the EndoBT-3987_WT_-Man_9_GlcNAc-2 crystal structure. **b** Two views of the electron density of Man_9_GlcNAc﻿ product shown at 1.0 σ r.m.s deviation. **c** Two views of the key residues of EndoBT-3987 interacting with Man_9_GlcNAc﻿  product are colored in orange. The catalytic residues are colored in yellow (D312 and E314). **d** Two views of superposition of Man_9_GlcNAc_2_Asn substrate (orange) and  Man_9_GlcNAc product (green).

The strategy for capturing a native binary enzyme-product complex was to perform co-crystallization experiments with the full-length EndoBT-3987 (EndoBT-3987_WT_) in the presence of Man_5_GlcNAc_2_Asn or Man_9_GlcNAc_2_Asn substrates. It worth noting that the enzyme was active against both HM *N*-glycan substrates. We thus obtained one snapshot of EndoBT-3987_WT_ in complex with the Man_5_GlcNAc product (EndoBT-3987_WT_-Man_5_GlcNAc; pdb code 6TCW), and two snapshots of the EndoBT-3987_WT_ in complex with the Man_9_GlcNAc product (EndoBT-3987_WT_-Man_9_GlcNAc-1, pdb code 6T8K; EndoBT-3987_WT_-Man_9_GlcNAc-2, pdb code 6T8I). EndoBT-3987_WT_-Man_5_GlcNAc crystallized in the *P* 2_1_ 2_1_ 2 space group with one molecule in the asymmetric unit and diffracted to a maximum resolution of 1.6 Å (Supplementary Table 1). Two crystal forms were obtained for the EndoBT-3987_WT_-GlcNAcMan_9_ complex, in space groups *P*1 (EndoBT-3987_WT_-Man_9_GlcNAc-1) and *P* 2_1_ 2_1_ 2_1_ (EndoBT-3987_WT_-Man_9_GlcNAc-2), and the corresponding crystal structures solved at 2.0 and 1.7 Å resolution, respectively (see Methods; Fig. 3; Supplementary Table 1; Supplementary Figs. 3 and 4). The overall protein scaffold and the conformation of the EndoBT-3987_WT_-Man_5_GlcNAc (r.m.s.d. of 0.35 Å for 397 residues), EndoBT-3987_WT_-Man_9_GlcNAc-1 (r.m.s.d. of 0.48 Å for 397 residues) and EndoBT-3987_WT_-Man_9_GlcNAc-1 (r.m.s.d. of 0.41 Å for 397 residues) crystal structures were essentially preserved with respect to the EndoBT-3987_D312A/E314L_-Man_9_GlcNAc_2_Asn substrate complex. We have decided to use the EndoBT-3987_WT_-Man_9_GlcNAc-2 crystal form for our description since it displays the highest resolution. In contrast to what is observed in the EndoBT-3987_D312A/E314L_-Man_9_GlcNAc_2_Asn complex, the C2-acetamide group of the GlcNAc (−1) residue is oriented towards its anomeric carbon in the EndoBT-3987_WT_-Man_9_GlcNAc-2 crystal structure. The N2 and O7 atoms of the acetamide group make hydrogen bonds with the side chain of D312 and Y380 (Fig. 3d). The boat ^1,4^*B* conformation of GlcNAc (−1) is further stabilized by hydrogen bond interactions between the O1 atom with the side chain of E314, the O3 atom with the side chain of Y315, and the O6 atom with the side chain of E401. The GlcNAc (−1) shows a ^1^*S*_5_ conformation in the EndoBT-3987_WT_-Man_5_GlcNAc crystal structure, but in this case a calcium cation is coordinated to O6 and O1 of GlcNAc (−1) (For more information about the catalytic cycle of EndoBT-3987, please see the Supplementary Note 1 and Supplementary Fig. 3).

### Structural basis of EndoBT-3987 specificity for HM glycans

**Fig. 4:**
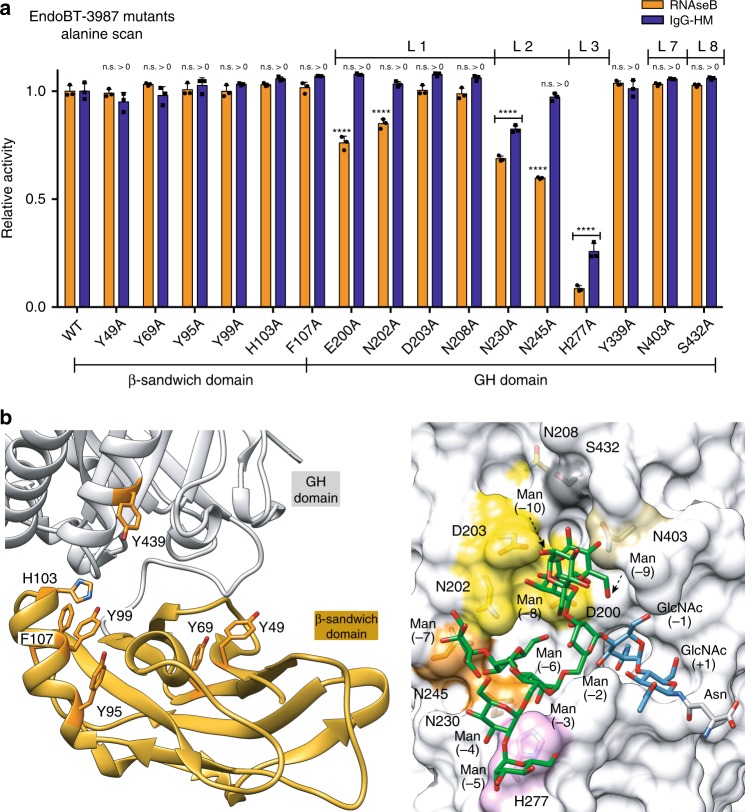
Alanine scan mutagenesis of EndoBT-3987 active site for HM IgG1 and RNaseB. **a** Hydrolytic activity of EndoBT-3987 and mutants against HM-IgG1 and RNaseB is shown, as determined by LC-MS analysis, normalized to EndoBT-3987_WT_. Statistical significance compared with wild-type EndoBT is annotated (multiple comparisons test, Tukey method; *p < 0.05; **p < 0.01; ***p < 0.001, ****p < 0.0001, n.s. > 0, not significantly greater than no-enzyme control). Source data are provided as a ‘Source_Data_File_Fig_4a’. **b** In the left panel cartoon representation of the β-sandwich domain. The mutated residues are highlighted in oranges. In the right panel surface representation of the EndoBT-3987_D312A/E314L_-Man_9_GlcNAc_2_Asn crystal structure showing the alanine mutations performed in loop 1 (yellow), loop 2 (orange), loop 3 (pink), loop 7 (brown), and loop 8 (gray) in the glycosidase domain (gray).

To further investigate how EndoBT-3987 specifically recognizes HM substrates at the molecular level, we performed single alanine mutations of residues in the loops that decorate the β-barrel core of the enzyme and contact the Man_9_GlcNAc_2_Asn substrate *N*-linked glycan. We studied their ability to process the *N*-linked HM glycans on two substrates, ribonuclease B (RNaseB) and HM-IgG. Specifically, we mutated key residues in loop 1 (E200, N202, D203, and N208), loop 2 (N230 and N245), loop 3 (H277A), loop 7 (Y403), and loop 8 (S432). As depicted in Fig. 4, H277A from loop 3 drastically reduced the hydrolytic activity against RNaseB and IgG of the enzyme. Furthermore, the N230A and N245A mutants located into the β-hairpin of loop 2, also reduced the activity of EndoBT-3987 on both substrates and E200A and N202A mutants located in loop 1 that interact with antenna 1b produced a smaller reduction of the hydrolytic activity of the enzyme. In contrast, mutations in loops 7 or 8 did not affect the activity of the enzyme against RNaseB nor IgG (Fig. 4). Collectively, the mutational analysis of the EndoBT-3987 loops that contact the HM glycan indicated that the interactions with the antennae 1b (loops 1, 2, and 3) were critical for glycan recognition, while those with antenna 2 (loops 1, 7, and 8) were effectively dispensable. Finally, we studied the influence of the β-sandwich domain into the hydrolytic activity of EndoBT-3987. Two deep cavities were identified at the interface between the β-sandwich and GH domains of the enzyme. We performed alanine mutations of residues in (i) cavity 1, located in β1 (Y49), α1 (Y95A, Y99A, and H103) and the α1-β5 loop (F107), and (ii) cavity 2, located in β3 (Y69). We also mutated Y439 to alanine in α7 of the GH domain, this residue points to the cavity one of the β-sandwich domain and could also be involved in carbohydrate binding. All mutant variants were enzymatically active as the wild-type enzyme, strongly suggesting that the two cavities are dispensable for the HM cleavage by EndoBT-3987.

## Discussion

**Fig. 5:**
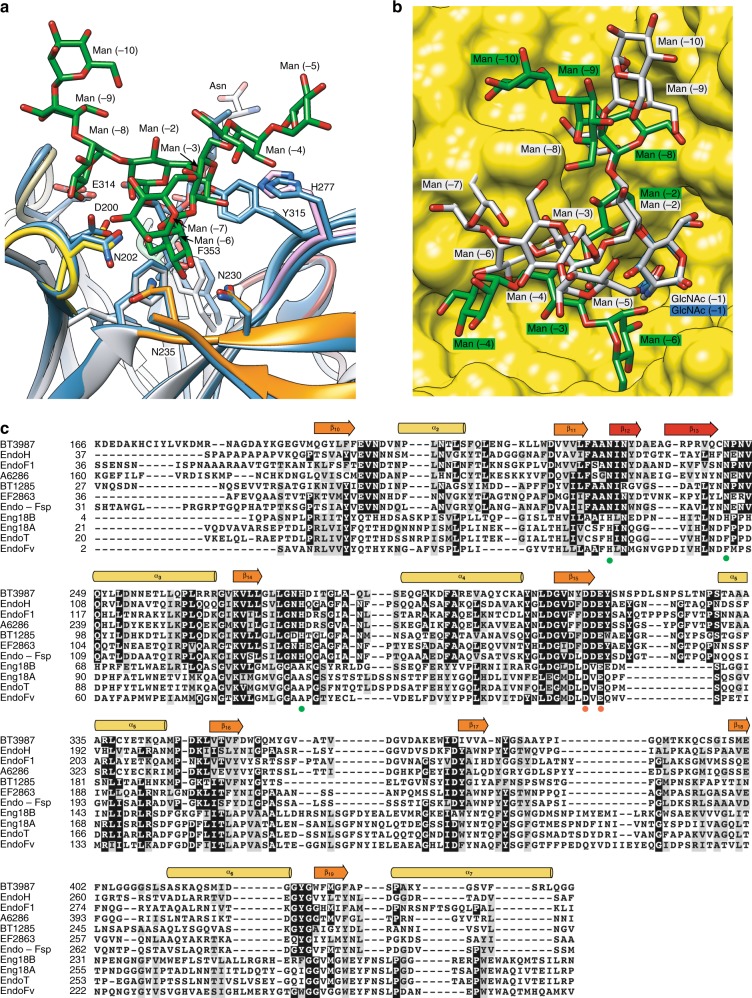
Structural basis of EndoBT-3987 specificity for HM-type *N*-glycans. **a** Structural comparison of EndoBT-3987_D312A/E314L_-Man_9_GlcNAc_2_Asn in gray and EndoH (PDB CODE 2EBN) in blue. The residues involved in substrate recognition are colored by loops: loop 1 (yellow), loop 2 (orange), and loop 3 (pinked). **b** Structural comparison of the HM-type glycan conformation in the active site of EndoBT-3987_WT_-Man_9_GlcNAc-2 (gray) and EndoS2 (PDB CODE 6MDV) (green) on surface representation of binding site of EndoS2 (yellow). **c** Structure weighted sequence alignment of BT3987 with GH18 ENGases family with characterized endo-*N*-acetyl-β-D-glucosaminidase activity against HM-type *N*-glycans and inactive against CT-type *N*-glycans. Comparison of BT3987 from *B. thetaiotaomicron* VPI-5482 (Q8A0N4, Uniprot code), EndoH from *Streptomyces plicatus* (P04067, Uniprot code), EndoF1 from *Elizabethkingia meningoseptica* (P36911, Uniprot code), A6286 from *Prevotella melaninogenica* (D9RSV7, Uniprot code), BT1285 from *B. thetaiotaomicron* VPI-5482 (Q8A889, Uniprot code), EF2863 from *Enterococcus faecalis* (Q830C5, Uniprot code), Endo-Fsp from Flavobacterium sp. (P80036, Uniprot code), Eng18B from *Hypocrea atroviride IMI 206040* (G9P8KO, Uniprot code), Eng18A from *Hypocrea atroviride IMI 206040* (G9NR36, Uniprot code), EndoT from *Hypocrea jecorina* (C4RA89, Uniprot code), and EndoFv from *Flammulina velutipe*s (D1GA49, Uniprot code). The catalytic residues are marked with red dots and the key residues that interact with Man_9_ are marked with green dots.

To further advance the understanding of EndoBT-3987 *N*-glycan specificity, we performed a structural analysis in the context of the GH18 family of ENGases. A search for structural homologs using the DALI server revealed that EndoBT-3987 shows significant structural similarity with EndoF1 from *E. meningoseptica* (pdb code 2EBN; Z-score of 37.5; r.m.s.d. value of 1.7 Å for 214 aligned residues; 42% identity)^48^, and EndoH from *Streptomyces plicatus* (pdb code 1C8Y; Z-score of 30.9; r.m.s.d. value of 2.4 Å for 214 aligned residues; 29% identity)^49^. EndoBT-3987, EndoF1, and EndoH are highly specific for HM and Hy-type *N*-glycans, but are able to process a broad range of proteins. In contrast to EndoBT-3987, both EndoF1 and EndoH do not display an additional β-sandwich domain. A detailed comparison of the EndoBT-3987_D312A/E314L_-Man_9_GlcNAc_2_Asn and EndoBT-3987_WT_-Man_9_GlcNAc-2 binary complexes with those of the unliganded forms crystal structures of EndoF1 and EndoH reveals that the key residues that interact with the HM glycan substrate and product in EndoBT-3987 are well conserved in EndoF1 and EndoH, supporting a common recognition mechanism (Fig. 5a). EndoBT-3987 residues N230 and N245 (loop 2) and H277A (loop 3) lie in equivalent positions as 48, 63, and 95 in EndoF1, and 48, 61, and 95 in EndoH. Supporting this notion, the minimum *N*-glycan structure that EndoH and EndoF1 are able to hydrolyze is the core Manα1–3Manα1–6Manβ1–4GlcNAcβ1–4GlcNAc, including Man (−6), Man (−3), Man (−2), GlcNAc (−1), and GlcNAc (+1)^50^. The O2 atom of Man (−3) makes a critical hydrogen bond with H277, whereas the O3 and O4 of Man (−6) makes important interactions with the side chains of N245 and N230, respectively. As a consequence, the Man (−6) residue is deeply buried and in close contact with the β-hairpin of loop 2.

The GH18 family of ENGases can be classified according to their *N*-glycan specificity into three main groups: (i) enzymes that hydrolyze CT-type *N*-glycans, (ii) enzymes that hydrolyze HM-type *N*-glycans, and (iii) enzymes that hydrolyze both CT- and HM-type *N*-glycans. Most members able to hydrolyze HM-type *N*-glycans also hydrolyze Hy-type *N*-glycans^30^. The *N*-glycan specificity of the GH18 family members is due to unique interactions between the loops that decorate the β-barrel core of the enzymes and the *N*-glycan chemical structures^27,51^. EndoBT-3987, EndoF1, and EndoH, which are classified in the first group, show the capacity to hydrolyze HM-type but not CT-type *N*-glycans^27^. In contrast, EndoS and EndoF3 are able to hydrolyze CT-type but not HM-type *N*-glycans. Binding of EndoS to CT-type *N*-glycans is predominantly driven by GH domain loops that make contacts with the α(1,3) antenna, a mechanism that is conserved in EndoS2^27,52^. In contrast, our experimental data clearly showed that the HM-hydrolyzing enzymes preferentially recognize the α(1,6) antenna 1b of HM-type *N*-glycans. In that sense, molecular docking calculations of a CT-type *N*-glycan into EndoBT-3987 revealed that the α(1,6) antenna makes clashes with loop 2 and 3 and cannot be accommodated into the binding grooves due to steric hindrance (Supplementary Fig. 3a). EndoS2 is able to hydrolyze CT- and HM-type *N*-glycans. The crystal structure of EndoS2 in complex with Man_9_GlcNAc and alanine scan mutagenesis experiments revealed that EndoS2 interacts mostly with the antenna α(1,3) of HM glycans and not with the antenna α(1,6) as EndoBT-3987 does. Supporting this notion, the conformations of the antennae α(1,3) of the HM- and CT-type *N*-glycan in the binding site of EndoS2 are very similar between them, but different from the conformation of HM-type glycans found in the binding pocket of EndoBT-3987 (Fig. 5b).

Amino-acid sequence alignment with other GH18 ENGases that are able to hydrolyze HM-type glycans revealed that the important residues for substrate binding N230, N245 and H277 are conserved in EndoH^37,53^, EndoF1^50^, A6286^26^, BT1285^26^, EF2863^54^, and Endo-Fsp^55^, while Eng18B^56^, Endo18A^56^, EndoT^57^, and EndoFv^58^ have other residues at these positions, a histidine, phenylalanine and alanine instead of N230, N245, and H277, respectively (Fig. 5c). The superposition of the EndoT and EndoBT-3987_D312A/E314L_-Man_9_GlcNAc_2_Asn crystal structures (Supplementary Fig. 5b) shows some clashes of Man (−7) in antenna α(1,6) with loop 2. This suggests that the mechanism of HM-type glycan recognition and the glycan specificity is different than EndoBT-3987 in this subgroup of enzymes that are able to hydrolyze HM-type *N*-glycans vs CT-type *N*-glycans.

Amino-acid sequence alignment revealed that EndoBT-3987 shows high degree of sequence identity with other putative GH18 ENGases from four families of the order *Bacteriodales*, from the phylum *Bacteroidetes*: *Bacteroidaceae (Bacteroides), Dysgonamonadaceae (Dysgonamodas), Prevotellaceae (Prevotella)*, and *Rikenellaceae (Allistipes)* (Supplementary Fig. 6, Supplementary Data 1). The catalytic residues D312 and E314 and the residues important for the binding of HM-type N230, N245, and H277 in EndoBT-3987 are also conserved along the species, suggesting a similar mechanism of mammalian HM-type *N*-glycans degradation in this species. Interestingly, all of them have at least one additional *N*-terminal β-sandwich domain, attached to the GH18 ENGases domain, with conserved hydrophobic residues in all the β-strands. In some of the putative enzymes, the β-sandwich domain is classified as a BACON (*Bacteroidetes*-Associated Carbohydrate-binding often *N*-terminal) domain. It has been suggested that this domain binds carbohydrate-containing molecules most likely mucins^59^. However, it has been shown that the β-sandwich domain of the GH BoGH5A from *Bacteroides ovatus*, also classified as a BACON domain, did not bind glycan or protein substrates^21^. Altogether, and taking into account our alanine scanning mutagenesis experiments where the activity of the enzyme was not affected by any of the mutants in the β-sandwich domain, the experimental data suggest that the β-sandwich domain is not directly involved in substrate binding or enzymatic activity. Instead, the function of the β-sandwich domain might be to distance the GH domain from the cell surface and confer additional mobility to the catalytic domain to hydrolyze the *N*-glycan^21^.

In summary, we have determined the molecular mechanism by which EndoBT-3987 from *B. thetaiotaomicron*, the enzyme that initiates the degradation/processing of mammalian HM-type *N*-glycans in the intestine, specifically recognizes these glycans at the atomic level. *B. thetaiotaomicron* hydrolyzes HM-type *N*-glycans from carbohydrates in the diet but also processes HM-type *N*-glycans present in mucins, playing a role in mediating immune responses^60^. We have presented a series of structural snapshots of the reaction center of the EndoBT-3987 from *B. thetaiotaomicron*. During this sequence of events, we visualize how the enzyme guides the substrate Man_9_GlcNAc_2_Asn into the reaction center where the hydrolytic reaction takes place, and unveil the mechanism of Man_9_GlcNAc product release. Based on these data, we have identified homologous enzymes in four families of the order *Bacteroidales*, strongly supporting the notion of a common mechanism in the degradation of mammalian HM-type *N*-glycan among these species. Finally, this substrate recognition mechanism is shared by other important HM-modifying enzymes of the GH18 family, like EndoH or EndoF1, that have been extensively used in glycan analysis for more than 50 years, and for which such a mechanism of substrate specificity was largely unknown. These findings will inform future efforts to engineer enzymes with customized glycan specificities.

## Methods

### Cloning, expression, and purification of EndoBT-3987

The pSpeedET vector encoding the EndoBT-3987 (*B. thetaiotaomicron* VPI-5482) gene was purchased from DNASU plasmid repository (https://dnasu.org/DNASU/Home.do). The recombinant EndoBT-3987_WT_ protein is depicted in the Supplementary Fig. 2. Single and double-point mutations (Supplementary Table 2) were made by using the FastCloning method^61^. Full sequences were confirmed by Genwiz (https://www.genewiz.com). *Escherichia coli* BL21(DE3) pLysS cells (Novagen) transformed with the corresponding plasmid were grown in 2000 ml of LB medium supplemented with 50 μg ml^−1^ kanamycin at 37 °C. When the culture reached an OD_600_ value of 0.6–0.8, the culture was incubated at 22 °C for 1 h. The expression of EndoBT-3987_WT_ was induced by adding 0.5 mM IPTG. After ca. 16 h at 22 °C, the cells were harvested by centrifugation at 5000 × *g* for 20 min at 4 °C and resuspended in 50 ml of 50 mM Tris-HCl, pH 7.5, 500 mM NaCl and 10% glycerol, containing protease inhibitors (Thermo Scientific™, A32955) and 2.5 µl of Benzonase (Merck, 71205; solution A). Cells were disrupted by sonication (12 cycles of 10 s pulses with 60 s cooling intervals between the pulses, and 60% of amplitude) at 4 °C, and the suspension was centrifuged at 10,000 × *g* for 10 min at 4 °C. The supernatant after being filtrated by 0.2 μm and then applied into a HisPur NiNTA column (1 ml, Thermo Scientific) equilibrated with 50 mM Tris-HCl, pH 7.5, 500 mM NaCl and 10% glycerol. The elution was performed with a linear gradient of 0 to 500 mM imidazole in 20 ml of solution A at 1 ml min^−1^. These were concentrated in an Amicon Ultra-15 centrifugal filter unit (Millipore) with a molecular cutoff of 10 kDa at 4000 × *g*. EndoBT was further purified by size-exclusion chromatography using a Superdex 200 10/300 GL column (GE Healthcare) equilibrated in PBS, pH 7.4. The eluted protein was stored at −80 °C. Further purification steps were needed for crystallogenesis experiments with EndoBT-3987_WT_ and EndoBT-3987_D312A/E314L_. The protein was dialyzed against 50 mM Tris-HCl pH 7.5, 150 mM NaCl, 2 mM DTT, and 500 mM EDTA (solution C), with TEV protease (1:20 ratio), overnight at 18 °C. The completeness of the enzymatic digestion reaction was confirmed by SDS-PAGE and the solution protein was then loaded into a Superdex 200 26/60 column (350 ml; GE Healthcare), equilibrated in 20 mM Tris-HCl pH 7.5, 50 mM NaCl. The eluted protein was concentrated at 10 mg mL^−1^ using an Amicon Ultra-15 centrifugal filter unit (Millipore) with a molecular cutoff of 30 kDa at 4000 × *g*.

### EndoBT-3987 crystallization and data collection

EndoBT-3987_WT_ was crystallized by mixing 0.25 µL of a protein solution at 10 mg ml^−1^ in 20 mM Tris-HCl pH 7.5, 50 mM NaCl with 0.25 µL of 200 mM sodium bromide, and 20% (w/v) PEG 3350. Crystals grew in 1–2 days. They were transferred to a cryo-protectant solution containing 20% glycerol and frozen under liquid nitrogen. Complete X-ray diffraction datasets were collected at the beamline BL13-XALOC (ALBA, Cerdanyola del Valles, Spain). EndoBT-3987_WT_ crystallized in the trigonal space group *P* 3_1_ 2 1 with one molecule in the asymmetric unit and diffracted to a maximum resolution of 1.4 Å (Supplementary Table 1). The EndoBT-3987_D312A/E314L_-Man_9_GlcNAc_2_Asn complex was crystallized by mixing 0.25 µL of a protein solution at 10 mg ml^−1^ in 20 mM Tris-HCl pH 7.5, 50 mM NaCl and 2.5 mM Man_9_GlcNAc_2_Asn, with 0.25 µL of 100 mM M MES pH 6, 50 mM CaCl_2_ and 10% (w/v) PEG 3350. They were transferred to a cryo-protectant solution containing 20% glycerol and frozen under liquid nitrogen. Complete X-ray diffraction of both datasets were collected at beamline I24 (Diamond Light source, Oxfordshire, UK). The EndoBT-3987_D312A/E314L_-Man_9_GlcNAc_2_Asn complex crystallized in the triclinic space group *R* 3 with one molecule in the asymmetric unit and diffracted to a maximum resolution of 1.3 Å (Supplementary Table 1). The EndoBT-3987_WT_-Man_5_GlcNAc complex was crystallized by mixing 0.25 µL of a protein solution at 10 mg ml^−1^ in 20 mM Tris-HCl pH 7.5, 50 mM NaCl and 2.5 mM Man_5_GlcNAc_2_Asn, with 0.25 µL of 100 mM MES pH 6.0, 200 mM CaCl_2_ and 20% (w/v) PEG 6000. Crystals grew in 1–2 days. They were transferred to a cryo-protectant solution containing 10% glycerol and frozen under liquid nitrogen. Complete X-ray diffraction datasets were collected at the beamline BL13-XALOC (ALBA, Cerdanyola del Valles, Spain). The EndoBT-3987_WT_-Man_5_GlcNAc complex crystallized in the orthorhombic space group *P* 2 2_1_ 2_1_ with one molecule in the asymmetric unit and diffracted to a maximum resolution of 1.6 Å (Supplementary Table 1). The EndoBT-3987_WT_-Man_9_GlcNAc complex was crystallized in two crystal forms, referred thereafter as EndoBT-3987_WT_-Man_9_GlcNAc-1 and EndoBT-3987_WT_-Man_9_GlcNAc-2. The first crystal form, EndoBT-3987_WT_-Man_9_GlcNAc-1, was obtained by mixing 0.25 µL of a protein solution at 10 mg ml^−1^ in 20 mM Tris-HCl pH 7.5, 50 mM NaCl and 2.5 mM Asn-GlcNAc_2_Man_9_ with 0.25 µL of 0.02 M sodium/potassium phosphate, 100 mM SPG (succinic acid, phosphate, glycine) system pH 9.0, and 25% (w/v) PEG 1500. The second crystal form EndoBT-3987_WT_-Man_9_GlcNAc-2 was obtained by mixing 0.25 µL of a protein solution at 10 mg ml^−1^ in 20 mM Tris-HCl pH 7.5, 50 mM NaCl and 2.5 mM Man_9_GlcNAc_2_Asn with 0.25 µL of 0.02 M sodium/potassium phosphate, 100 mM Bis-Tris propane pH 8.5, and 20% (w/v) PEG 3350. Both crystal forms grew in 1–2 days. They were transferred to a cryo-protectant solution containing 10% glycerol and frozen under liquid nitrogen. Complete X-ray diffraction datasets for both crystal forms were collected at beamline I03 (Diamond Light source, Oxfordshire, UK). EndoBT-3987_WT_-Man_9_GlcNAc-1 crystal and crystallized in the triclinic space group *P*1 with two molecules in the asymmetric unit and diffracted to a maximum resolution of 2.0 Å (Supplementary Table 1). The second crystal form EndoBT-3987_WT_-Man_9_GlcNAc-2, crystallized in the orthorhombic space group *P* 2_1_ 2_1_ 2_1_ with one molecule in the asymmetric unit and diffracted to a maximum resolution of 1.7 Å (Supplementary Table 1). All datasets were integrated and scaled with XDS following standard procedures^62^.

### EndoBT-3987 structures determination and refinement

Structure determination of EndoBT-3987_WT_ was carried out by molecular replacement methods implemented in Phaser^63^ and the PHENIX suite^64^, and using the pdb code 3POH as a search template. Structure determination of EndoBT-3987_D312A/E314L_-Man_9_GlcNAc_2_Asn, EndoBT-3987_WT_-Man_5_GlcNAc, EndoBT-3987_WT_-Man_9_GlcNAc-1, and EndoBT-3987_WT_-Man_9_GlcNAc-2 complexes were carried out by molecular replacement using the crystal structure of EndoBT-3987_WT_ as a template model. Model rebuilding was carried out with Buccaneer^65^ and the *CCP4* suite^66^. The final manual building was performed with Coot^67^ and refinement with phenix.refine^68^. The structure was validated by MolProbity^69^. Data collection and refinement statistics are presented in Supplementary Table 1. Molecular graphics and structural analyses were performed with the UCSF Chimera package^70^.

### Chemoenzymatic preparation of HM *N*-glycans

The HM *N*-linked glycan Man_9_GlcNAc_2_Asn was prepared by digestion of soybean agglutinin isolated from soybean flour, and subsequent chromatographic purification. Crude soybean agglutinin (3.2 g) was obtained from 500 g of soybean flour (Sigma) through fractional precipitation with ammonium sulfate and digested thoroughly with pronase (Sigma). The digestion was filtered, and the filtrate was lyophilized. The residue was loaded onto a column (1.5 × 70 cm) of Sephadex G50 (Sigma), which was pre-equilibrated and eluted with 0.1 M AcOH. The fractions containing Man_9_GlcNAc_2_Asn were pooled and lyophilized. The material was finally purified by reverse-phase HPLC to afford homogeneous Man_9_GlcNAc_2_Asn (20 mg) as a white powder after lyophilization, which was characterized by compositional analysis, HPLC, and electron spray ionization mass spectrometry (ESI-MS)^71^. ESI-MS: calculated for Man_9_GlcNAc_2_Asn, M = 1996.69 Da; found (*m/z*), 999.74 [M + 2H]^2+^, 685.98 [M + H + Na + K]^3+^. The Man_5_GlcNAc_2_Asn N-glycan was prepared by digestion of the high-mannose *N*-glycan (Man_9_GlcNAc_2_Asn) using the α1,2-mannosidase from *B. thetaiotaomicron* (Supplementary Fig. 7)^72^. Specially, the mannosidase (10 μg) was added to a solution of Man_9_GlcNAc_2_Asn (10 mg, 0.005 mmol) in a buffer (PBS, 100 mM, pH 7.4, 1 mL). The reaction was monitored with LC-MS (Waters), and the desired product was purified with size-exclusion chromatography (GE Healthcare) to give the pure Man_5_GlcNAc_2_Asn N-glycan as a white powder after lyophilizing (6.34 mg, 94%). ESI-MS: calcd for Man_5_GlcNAc_2_Asn, M = 1349.21 Da; found (*m*/*z*), 675.59 [M + 2H]^2+^, 1349.90 [M + H]^+^.

### Purification of high-mannose Fc and IgG1

CD4-induced (CD4i) IgG1 plasmid^73^ was expressed in HEK293T cells (ATCC) using polyethyleneimine as a transfection agent. Kifunensine, a potent inhibitor of the mannosidase I enzyme, was used to ensure HM glycans were present on the Fc and IgG1^44^. After transfection, cells were cultured for 96 h in Free-style F17 medium supplemented with GlutaMAX and Geneticin (Thermo Fisher Scientific). HM IgG1 was purified using protein A chromatography, with 20 mM sodium phosphate buffer pH 7.0 used as a binding buffer and 100 mM sodium citrate buffer pH 3.0 as elution buffer. The fractions were neutralized with 1 M Tris pH 9.0. SDS-PAGE was used to identify fractions which contained HM IgG1. These were subsequently pooled and concentrated.

### Enzymatic activity assays of alanine EndoBT-3987 mutants

Reactions for the alanine scan mutants were set up using 100 nM EndoBT-3987_WT_ for reactions with RNAseB and 500 nM for reactions with high-mannose IgG1. The enzymes were mixed with 5 μM substrate in PBS pH 7.4 at room temperature. For the alanine scan, 10 μl aliquots of the reaction were taken in triplicate and allowed to progress for 30 and 45 min for RNAseB and high-mannose IgG1 substrates, respectively. All reactions were quenched using 1.1 μL of 1% trifluoroacetic acid. The quenched reactions were then mixed with 50 mM TCEP and analyzed by LC-MS using an Accela LC System attached to a LXQ linear ion trap mass spectrometer (Thermo Scientific, Waltham, MA). Relative amounts of the substrate and hydrolysis products were quantified after deconvolution of the raw data and identification of the corresponding peaks using BioWorks (Thermo Scientific, Waltham, MA). The data were plotted and statistical significance was determined using a multiple comparisons test (Tukey method) in GraphPad (GraphPad Software, LaJolla, CA).

### Reporting summary

Further information on research design is available in the Nature Research Reporting Summary linked to this article.

## Supplementary information

Supplementary Information

Description of Additional Supplementary Files

Supplementary Data 1

Reporting Summary

## Data Availability

The atomic coordinates and structure factors have been deposited with the Protein Data Bank, accession codes 6T8I (EndoBT-3987_WT_), 6TCV (EndoBT-3987_D312A/E314L_-Man_9_GlcNAc_2_Asn), 6TCW (EndoBT-3987_WT_-Man_5_GlcNAc), 6T8K (EndoBT-3987_WT_-Man_9_GlcNAc-1), and 6T8L (EndoBT-3987_WT_-Man_9_GlcNAc-2). The source data underlying Fig. 4a and Supplementary Fig. 1b are provided as a Source Data file. Other data are available from the corresponding authors upon reasonable request.

## Source data

Source Data
