# Supplementary material for: Structural basis of mammalian high-mannose N-glycan processing by human gut Bacteroides
Source: Nat Commun. 2020 Feb 14;11:899. doi: 10.1038/s41467-020-14754-7 (PMC7021837; doi:10.1038/s41467-020-14754-7)
Supplement: Supplementary file 1 — Supplementary Information [file 41467_2020_14754_MOESM1_ESM.pdf]

SUPPLEMENTARY INFORMATION

Structural basis of high-mannose mammalian N-glycan processing by human gut *Bacteroides*.

Trastoy *et al.*

SUPPLEMENTARY TABLES

Supplementary Table 1. Data collection and refinement statistics

	EndoBT-3987$_{WT}$	EndoBT-3987$_{D312A/E314L}$-Man$_9$GlcNAc$_2$Asn	EndoBT-3987$_{WT}$-Man$_5$GlcNAc	EndoBT-3987$_{WT}$-Man$_9$GlcNAc-1	EndoBT-3987$_{WT}$-Man$_9$GlcNAc-2
**PDB code**	**6T8I**	**6TCV**	**6TCW**	**6T8K**	**6T8L**
**Beamline**	BL13-XALOC	I24 (DLS)	BL13-XALOC	I03 (DLS)	I03 (DLS)
**Wavelength (Å)**	0.9791	0.9791	0.9791	0.9762	0.9762
**Resolution range (Å)**	46.42-1.4	28.87- 1.31	20 -1.6	29.28- 2.0	19.18 -1.7 (1.761 - 1.7)
**Space group**	P 3_1 2 1	R 3 :H	P 21 21 2	P 1	P 21 21 21
**Unit cell**	74.6, 90, 74.6, 90, 133.5, 120	115.5, 115.5, 97.4, 90, 90, 120	76.4, 133.6, 49.7, 90, 90, 90	46.9, 59.6, 76.1, 97.3, 90.1, 92.3	49.4, 76.3, 116.7, 90, 90, 90
**Total reflections**	831752 (82754)	566888 (46520)	434692 (44512)	96359 (9648)	323857 (32614)
**Unique reflections**	85310 (8417)	115141 (10639)	66165 (6469)	53616 (3567)	49301 (4854)
**Multiplicity**	9.8 (9.9)	4.9 (4.4)	6.6 (6.9)	1.8 (1.8)	6.6 (6.7)
**Completeness (%)**	99.9 (99.9)	99.10 (92.02)	98.10 (97.51)	93.27 (64.38)	99.96 (99.98)
**Mean I/sigma(I)**	24.5 (3.7)	10.75 (1.33)	15.42 (2.82)	9.89 (3.75)	16.46 (3.44)
**Wilson B-factor**	16.6	15.8	17.95	19.0	15.2
**R-merge**	0.044 (0.528)	0.068(0.659)	0.0784 (0.653)	0.054 (0.217)	0.080 (0.482)
**CC1/2**	1 (0.942)	0.998 (0.671)	0.998 (0.895)	0.991 (0.775)	0.999 (0.903)
**CC***	1 (0.985)	0.999 (0.896)	1 (0.972)	0.998 (0.935)	0.999 (0.903)
**Reflections used in refinement**	85229 (8415)	115139 (10638)	66157 (6469)	51477 (3558)	49298 (4853)
**Reflections used for R-free**	4246 (393)	5621 (484)	3309 (324)	2419 (178)	2502 (235)
**R-work**	0.16 (0.19)	0.1587 (0.23)	0.1727 (0.24)	0.1618 (0.19)	0.15 (0.18)
**R-free**	0.18 (0.21)	0.1772 (0.24)	0.1893 (0.28)	0.1965 (0.25)	0.18 (0.26)
**Number of non-H atoms**	3692	3511	3734	7533	3837
**Macromolecules**	3409	3110	3356	6796	3347
**Ligands**	12	133	78	230	115
**Protein residues**	434	397	428	507	432
**RMS(bonds, Å)**	0.005	0.014	0.016	0.01	0.018
**RMS(angles, º)**	0.82	1.37	1.71	1.49	1.8
**Ramachandran favored (%)**	97.9	97.4	97.4	97.6	97.9
**Ramachandran allowed (%)**	2.1	2.6	2.6	2.3	2.1
**Ramachandran outliers (%)**	0	0.0	0.00	0.00	0.00
**Rotamer outliers (%)**	0.5	0.9	1.1	0.8	0.6
**Clashscore**	0.7	1.7	2.1	1.6	0.7
**Average B-factor ($Å^2$)**	22.6	21.1	23.1	21.4	18.4
**Macromolecules**	22.9	20.4	22.4	21.0	17.3
**Ligands**	23.8	21.6	23.6	23.6	25.3
**Solvent**	31.1	28.2	31.4	24.8	26.8

Statistics for the highest-resolution shell are shown in parentheses

Supplementary Table 2. Primer sequences used in EndoBT-3987 cloning experiments

EndoBT Mutant	Forward Primer(5'-3')	Reverse Primer (5'-3')
Y49A	ctttccatcacgcagagctgcattattttcataaatgccactgtaggc	gcctacagtggcatttatgaaaataatgcagctctgcgtgatggaaag
Y69A	tactgtagtagcagcagtctcaccgtgcaattcgacaacc	ggttgtcgaattgcacggtgagactgctgctactacagta
Y95A	tgcgccttattatacgtttccaaagcagcagcatcaattttcacttttgc	gcaaaagtgaaaattgatgctgctgctttggaaacgtataataaggcgca
Y99A	caaaatctgtattatgcgccttattagccgtttccaaataagcagcatcaattt	aaattgatgctgcttatttggaaacggctaataaggcgcataatacagattttg
H103A	cggatacaatgcaaaatctgtattagccgccttattatacgtttccaaataa	ttatttggaaacgtataataaggcggctaatacagattttgcattgtatccg
F107A	agtcctgcggatacaatgcagcatctgtattatgcgccttattatac	gtataataaggcgcataatacagatgctgcattgtatccgcaggact
E200A	taatgcaaggttatttgttctttgcagtaaatgacgtaaatccgctg	cagcggatttacgtcatttactgcaaagaacaaataaccttgcatta
N202A	ttcagcggatttacgtcagctacttcaaagaacaaataaccttgcattacac	gtgtaatgcaaggttatttgttctttgaagtagctgacgtaaatccgctgaa
D203A	gttatttgttctttgaagtaaatgccgtaaatccgctgaatactctttc	gaaagagtattcagcggatttacggcatttacttcaaagaacaaataac
N208A	attctctaattggaaagaaagagtagccagcggatttacgtcatttacttca	tgaagtaaatgacgtaaatccgctggctactctttctttccaattagagaat
N230A	atgttgttgtactgtttgctgctgctatcaactatgatgctgaagcag	ctgcttcagcatcatagttgatagcagcagcaaacagtacaacaacat
N245A	ctgtacattcggggcacattgtacacgaggacgtcctgc	gcaggacgtcctcgtgtacaatgtgccccgaatgtacag
H277A	tgccaagcctgtaatgtcagcgttaccaagcagtcctaac	gttaggactgcttggtaacgctgacattacaggcttggca
Y339A	ctgtttggtttcagcacacagacgagctgcagcagcc	ggctgctgcagctcgtctgtgtgctgaaaccaaacag
N403A	actaccgccaccacctaaggcgaattccattgaaataccgga	tccggtatttcaatggaattcgccttaggtggtggcggtagt
S432A	catatttagccggagctggagcaaatcccataaaccatccat	atggatggtttatgggatttgctccagctccggctaaatatg

SUPPLEMENTARY NOTE 1

The catalytic cycle of EndoBT-3987

We are now able to visualize two steps of the catalytic cycle of EndoBT-3987: (i) the binding of the *N*-linked $Man_9GlcNAc_2Asn$ substrate to the active site of the enzyme and (ii) the binding of the glycan products, $Man_5GlcNAc$ and $Man_9GlcNAc$, before being released from the active site (SI Fig. 3a). The proposed substrate-mediated catalytic mechanism has been extensively studied for chitinases (EC 3.2.1.14) of the GH18 family, and involves the participation of the 2-acetamide group of the substrate GlcNAc (-1).[1–5] In the first step, the *N*-linked HM glycan substrate binds to the active site generating the distortion of the GlcNAc (-1) residue from a more energetic favorable chair $^{1}C_{4}$ conformation, to a skew boat $^{1}S_{5}$ conformation. The E314 residue acts in this step as an acid and protonates the glycosidic bond. The D312 residue makes a hydrogen bond with the nitrogen of the C2-acetamide group of GlcNAc (-1). Thus, the carbonyl group attacks the anomeric carbon of GlcNAc (-1) leading to the formation of an oxazolinium intermediate.[1,2,6,7] Recently, QM/MM metadynamics simulations on chitinase B from *Serratia marcescens (Sm*ChiB*)* suggests that this reaction intermediate is a neutral oxazoline with a oxazolinium ion formed towards the reaction products.[8] In the EndoBT-$3987_{D312A/E314L}$-$Man_9GlcNAc_2Asn$ substrate complex, GlcNAc (-1) is in a skew boat $^{1}S_{5}$ conformation but the C2-acetamide group is orientated towards A312, stabilized by hydrogen bonds between the nitrogen of this group and Y380, and it is not able to reach the anomeric carbon. The oxygen of the acetamide group requires D312 to rotate 180 º and produce the first step of the reaction.[2] In the second step, E314 acts as a base and deprotonates a water molecule that attacks the anomeric carbon of GlcNAc (-1), breaking the oxazoline ring and regenerating the hemiacetal sugar with retention of anomeric configuration (SI Fig. 3b;[1]). The skew-boat conformation $^{1}S_{5}$ is also observed in other x-ray crystal structures of chitinases in complex with the glycan substrate where the acid/base glutamic acid residue is mutated by a leucine of GH18

family. [9,10] However, GlcNAc (-1) adopts a boat conformation $^{1,4}B$ in x-ray crystal structures of chitinases in which the acid/base residue is mutated by glutamine (SI Fig. 3c). [1,11] We have mutated the acid/base residue by a hydrophobic residue in order to completely abolish the activity of the enzyme, because by working with other GH18 ENGases we have observed some residual hydrolytic that we would like to avoid in our co-crystallization experiments. Enzymes from the GH84 and GH20 families also show a substrate mediated mechanism. In the crystal structures of enzyme-substrates complexes from GH84 and GH20 families, GlcNAc (-1) adopts $^{1,4}B/^{4}E$[12] and $^{1}S_{3}$[13] conformations, respectively. It is worth noting that, in the enzyme-$Man_9GlcNAc$ structures, GlcNAc (-1) displays a $^{1,4}B$ conformation, as observed in other chitinases.[14–17] However, GlcNAc (-1) shows a $^{1}S_{5}$ conformation in the EndoBT-3987$_{WT}$-$Man_5GlcNAc$ crystal structure, but in this case a calcium cation is coordinated to O6 and O1 of GlcNAc (-1). The crystal structure of EndoS2, another GH18 ENGase, in complex with the $Man_9GlcNAc$ product, GlcNAc (-1) also displayed a $^{1}S_{5}$ conformation[18], a conformation also proposed by QM/MM metadynamics simulations on *Sm*ChiB[8]. The boat conformation $^{1,4}B$ and the skew boat $^{1}S_{5}$ conformation are close in the conformational landscape for pyranoses.[19]

This mechanism requires the highly conserved D_1XD_2XE catalytic motif in the family that performs the double displacement reaction. The role of D_1 is to provide a negative charge that keeps D2(D312)-E(E314) protonated[1,4]. It has been shown that the entrance of the substrate produces the rotation of D_2, stabilized by D_1 in the unliganded enzyme, to form hydrogen bonds with E. In EndoBT-3987 D_1 is substituted by an asparagine, and we did not observe any conformational changes in D312 from the unliganded to the enzyme-product structures. However, in both unliganded X-ray crystal structures of EndoBT$_{WT}$ (pdb codes 6T8I and 3POH), the conformation of the acid/base residue E314 is facing Y315, making hydrogen bonds with a water molecule and too far away from D312 to make hydrogen bonds ($D312_{OD1}$-$E314_{OE2}$ = 4.52 Å). This conformation is different in the enzyme-product complex structures where E314

is at a distance that can interact with D312 ($D312_{OD1}$-$E314_{OE2}$ = 2.55 Å) by hydrogen bonds. E314 is also closer to the O1 of GlcNAc (-1) (Glu_{OE2}-GlcNAc$(-1)_{O1}$ = 2.47 Å) that is protonated in the first reaction event in the enzyme-product complex structures. Thus, this might be the active conformation of E314 in the wild type enzyme-substrate complex in order to form hydrogen bonds with D312[1,8], protonate O4 of GlcNAc (+1), and later deprotonate a water molecule that produces the second nucleophilic attack on the anomeric carbon. In addition, the conformation of F353 changes when the substrate enters the binding site in order to accommodate GlcNAc (-1) and GlcNAc (+1). This conformation remains after Asn-GlcNAc (+1) is released.

SUPPLEMENTARY FIGURES

a

GDDLEVGKNIDESAYSGIYENNAYLRDGKSNLVSKVVELHGETYATTVKMGLSKTPNTATSAKV

KIDAAYLETYNKAHNTDFALYPQDLVTFANEGILTVNANTKSAEVEMTIRAGEGLQEDKTYAIP

VAISDQSSDITIKDEDAKHCIYLVKDMRNAGDAYKGEGVMQGYLFFEVNDVNPLNTLSFQLENG

KLLWDVVVLFAANINYDAEAGRPRVQCNPNVQYLLDNNETLLQPLRRRGVKVLLGLLGNHDITG

LAQLSEQGAKDFAREVAQYCKAYNLDGVNYDDEYSNSPDLSNPSLTNPSTAAAARLCYETKQAM

PDKLVTVFDWGQMYGVATVDGVDAKEWIDIVVANYGSAAYPIGQMTKKQCSGISMEFNLGGGGS

LSASKAQSMIDGGYGWFMGFAPSPAKYGSVFSRLQGGGEVLYGSNVAAPTIFYKKNDPTPYKYP

DDL

b

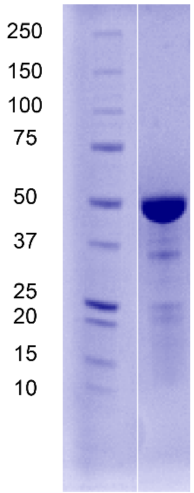

Supplementary Figure 1 | Recombinant production of EndoBT-3987. a The recombinant EndoBT-3987 construct (residues 27-476) highlighted in yellow. Residues not included in the sequences are highlighted in oranges. Catalytic residues are highlight in green. To obtain catalytic inactive EndoBT D312 and E314 were mutated by alanine and leucine, respectively. **b** SDS-PAGE showing purified EndoBT-3987. Source data is provided as a 'Source_data_file_Supplementary_Fig_1b'.

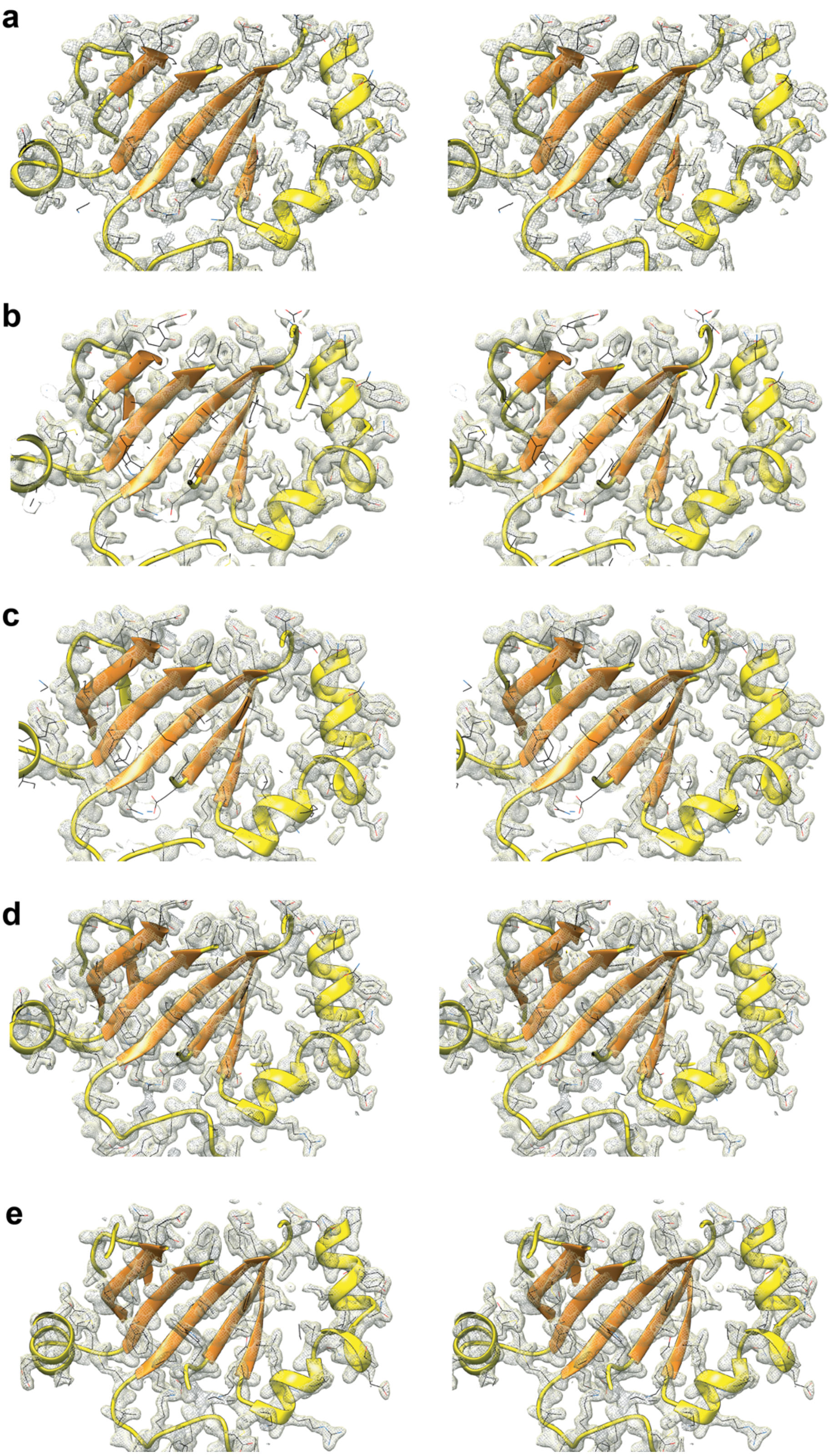

Supplementary Figure 2 | Electron density map of the refined EndoBT-3987 X-ray crystal structures. Stereo view of the final electron density maps (2mFo-DFc contoured at 1σ) corresponding to the EndoBT-3987$_{WT}$ (**a**), EndoBT-3987$_{D312A/E314L}$-$Man_9GlcNAc_2Asn$ (**b**), EndoBT-3987$_{WT}$-$Man_5GlcNAc$ (**c**), EndoBT-3987$_{WT}$-$Man_9GlcNAc$-1 (**d**), and EndoBT-3987$_{WT}$-$Man_9GlcNAc$-2 (**e**) structures.

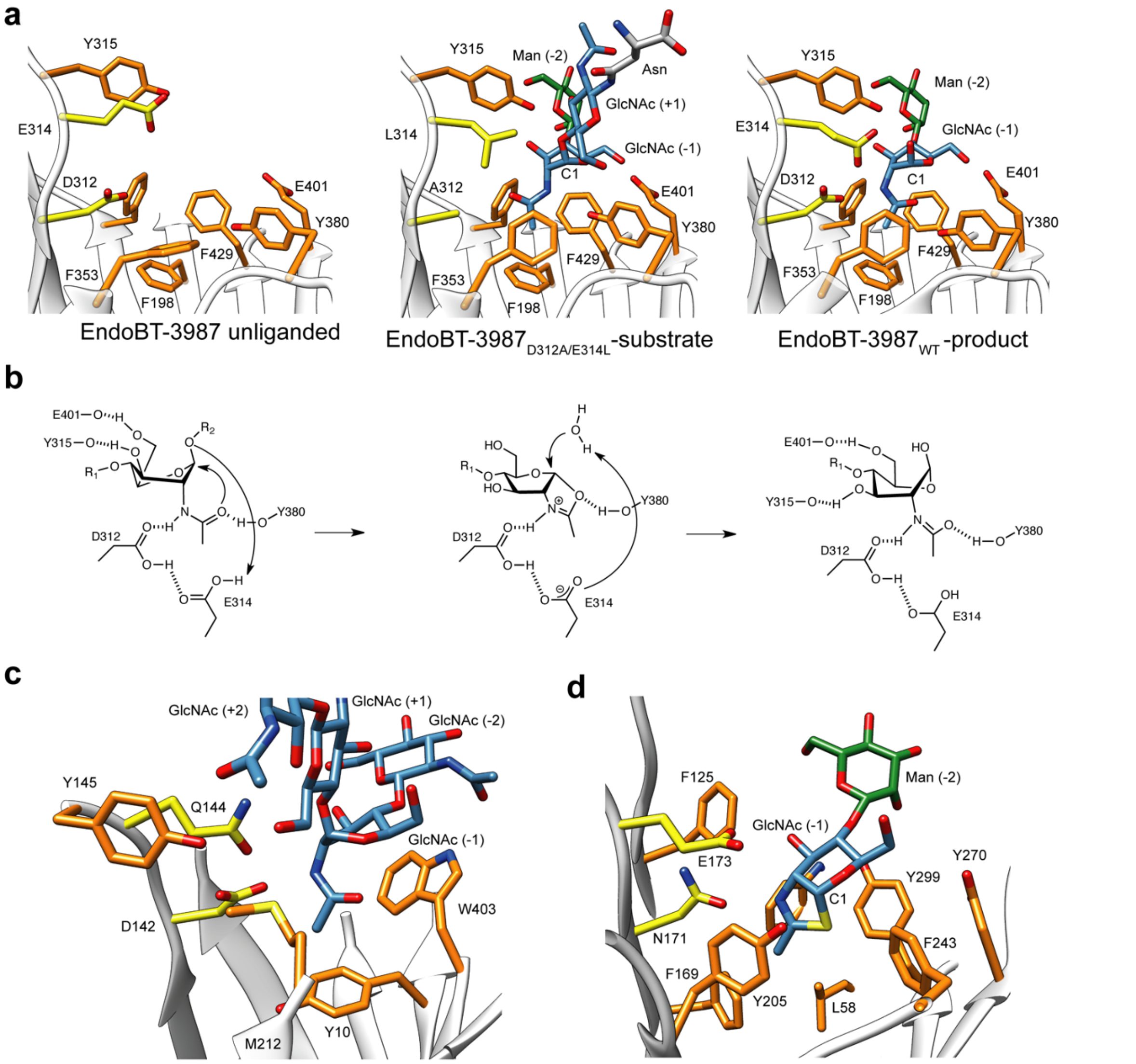

Supplementary Figure 3 | The catalytic mechanism of EndoBT-3987. a Catalytic site of EndoBT-3987 in the BT-3987-unliganded (left), EndoBT-3987$_{D312A/E314L}$-Man$_9$GlcNAc$_2$Asn (center) and EndoBT-3987$_{WT}$-Man$_9$GlcNAc-2 (right) crystal structures. In the first step, the *N*-linked HM glycan substrate binds to the active site generating the distortion of the GlcNAc (-1) residue from a more energetic favorable chair $^1C_4$ conformation, to a skew boat $^1S_5$ conformation. The E314 residue acts in this step as an acid and protonates the glycosidic bond. The D312 residue makes a hydrogen bond with the nitrogen of the C2-acetamide group of GlcNAc (-1). Thus, the carbonyl group attacks the anomeric carbon of GlcNAc (-1) leading to the formation of an oxazolinium intermediate. In the second step, E314 acts as a base and deprotonates a water molecule that attacks the anomeric carbon of GlcNAc (-1), breaking the oxazoline ring and regenerating the hemiacetal sugar with retention of anomeric configuration. **b** Chemical structure and symbol representation of Asn-Man$_9$ (upper panel) and proposed catalytic mechanism for EndoBT-3987. **c** x-ray crystal structure Chitinase B from Serratia marcescense inactive mutant E144Q in complex with N-acetylglucosamine-pentamer (GH18 chitinase) (pdb code: 1E6N). **d** EndoA from Arthrobacter protophormiae in complex with Man3GlcNAc-thiazoline (GH85 ENGase) (pdb code: 3FHQ).

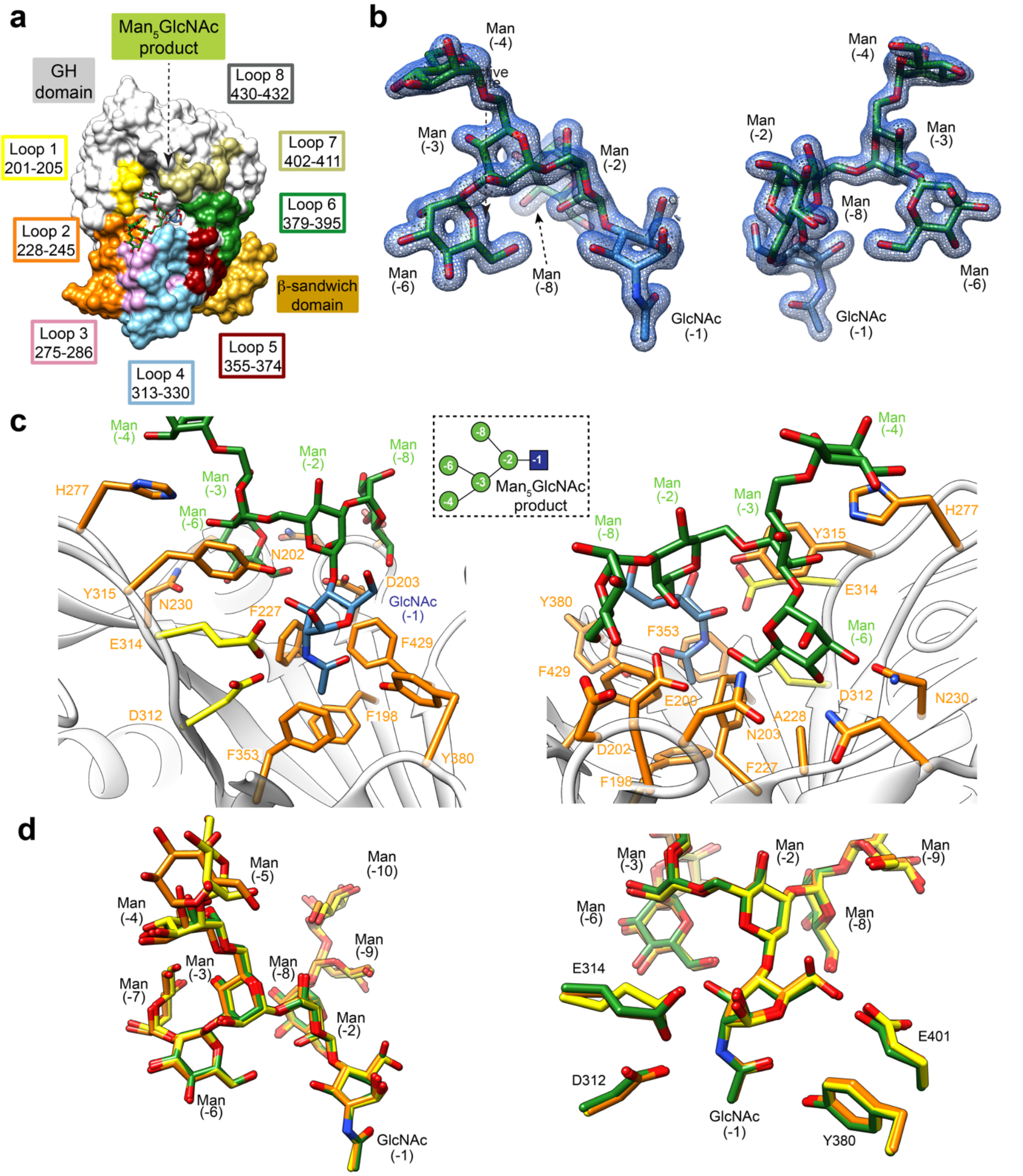

Supplementary Figure 4 | The product Man_5 glycan binding site. a Surface representation with annotated domains and GH loops of the EndoBT-3987$_{WT}$- $Man_5GlcNAc$ crystal structure. **b** Two views of the electron density of $Man_5GlcNAc$ product shown at 1.0 σ r.m.s deviation. **c** Two views of the key residues of EndoBT-3987 interacting with $Man_5GlcNAc$ substrate are coloured in orange. The catalytic residues are coloured in yellow (D312 and E314). **d** Two views of superposition of EndoBT-3987$_{WT}$- $Man_5GlcNAc$ product (green), EndoBT-3987$_{WT}$- $Man_9GlcNAc$-1 product (yellow), and EndoBT-3987$_{WT}$- $Man_9GlcNAc$-2 crystal structures (orange).

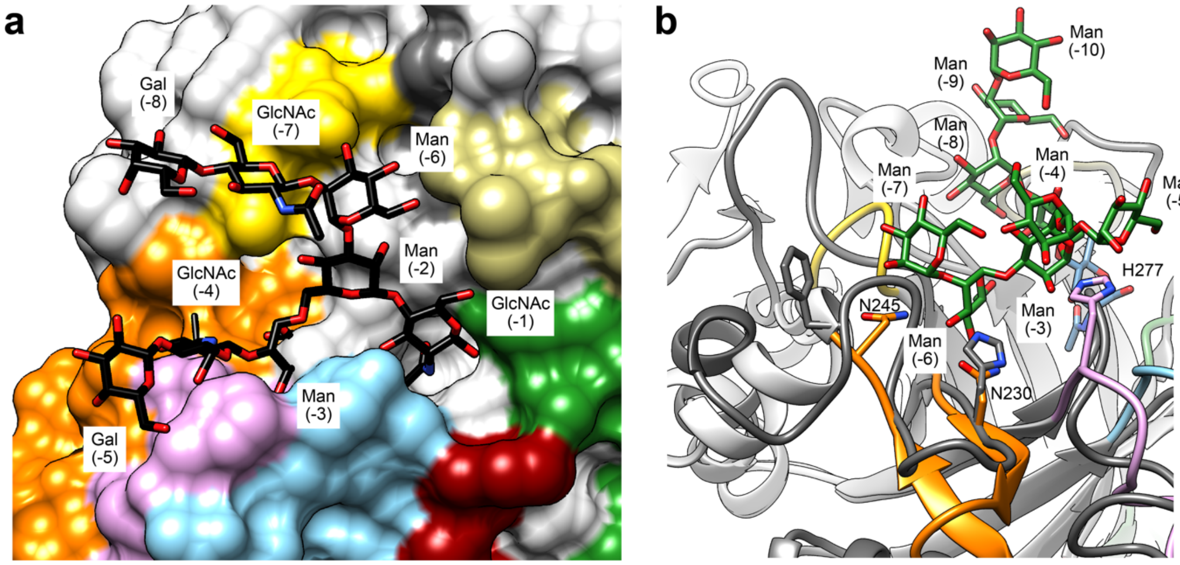

Supplementary Figure 5 | Structural basis of EndoBT-3987 specificity for high mannose type *N*-glycans. a Superimposition of EndoBT-3987_{WT} crystal structure and the CT-type glycan product from the EndoS2-CT crystal structure (pdb code: 6MDV). **b** Superimposition of EndoT crystal structure (pdb code: 4AC1) and the $Man_9GlcNAc$ product form the EndoBT-3987_{WT}- $Man_9GlcNAc$-2 crystal structure.

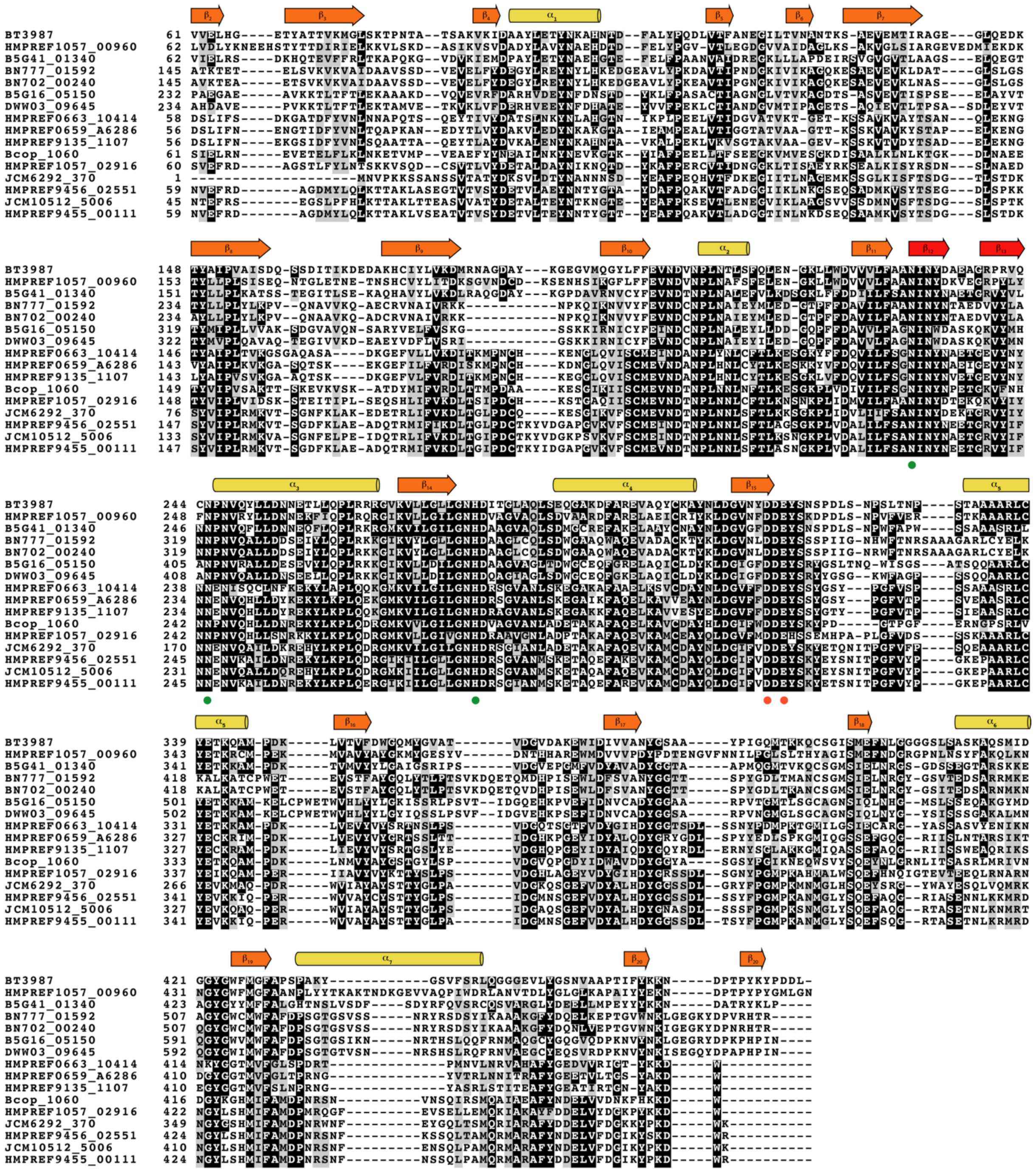

Supplementary Figure 6 | Sequence alignment of EndoBT-3987 with homologues found in the *Bacteroidetes* phylum. Comparison of BT3987 from *B. thetaiotaomicron VPI-5482* (Q8A0N4, Uniprot code), HMPREF1057_00960 from *B. finegoldii* CL09T03C10 (K5CRT6, Uniprot code, Identity: 49.3%), B5G41_01340 from *Alistipes onderdonkii* (A0A1Y3R5N0, Uniprot code, Identity: 46.8%), BN777_01592 from *Bacteroides sp. CAG:770* (R6TYV2, Uniprot code, Identity: 46.3%), BN702_00240 from *Bacteroides sp. CAG:545* (R5SGN4, Uniprot code), B5G16_05150 from Alistipes sp. An66 (A0A1Y3VE51, Uniprot code, Identity: 39.9%), DWW03_09645 from *Alistipes sp. AF14-19* (A0A374A074, Uniprot code, Identity: 38.6%), HMPREF0663_10414 from *Prevotella oralis ATCC 33269* (E7RMR4, Uniprot code, Identity: 35.9%), HMPREF0659_A6286 from *Prevotella melaninogenica strain ATCC 25845* (D9RSV7, Uniprot code, Identity: 35.9%), HMPREF9135_1107 from *Prevotella baroniae*

F0067 (U2QH48, Uniprot code, Identity: 35.6%), Bcop_1060 from *B. coprosuis DSM 18011* (F3ZTN6, Uniprot code, Identity: 36.5%), HMPREF1057_02916 from *B. finegoldii CL09T03C10* (K5CJA5, Uniprot code, Identity: 36%), JCM6292_370 from *B. pyogenes JCM 6292* (W4P4D8, Uniprot code, Identity: 39.1%), HMPREF9456_02551 from *Dysgonomonas mossii DSM 22836* (F8X2F1, Uniprot code, Identity: 34.7%), CM10512_5006 from *Bacteroides reticulotermitis JCM 10512* (W4V122, Uniprot code, Identity: 34.1%), HMPREF9455_00111 from *Dysgonomonas gadei ATCC BAA-286* (F5ISP4, Uniprot code, Identity: 34.7%).

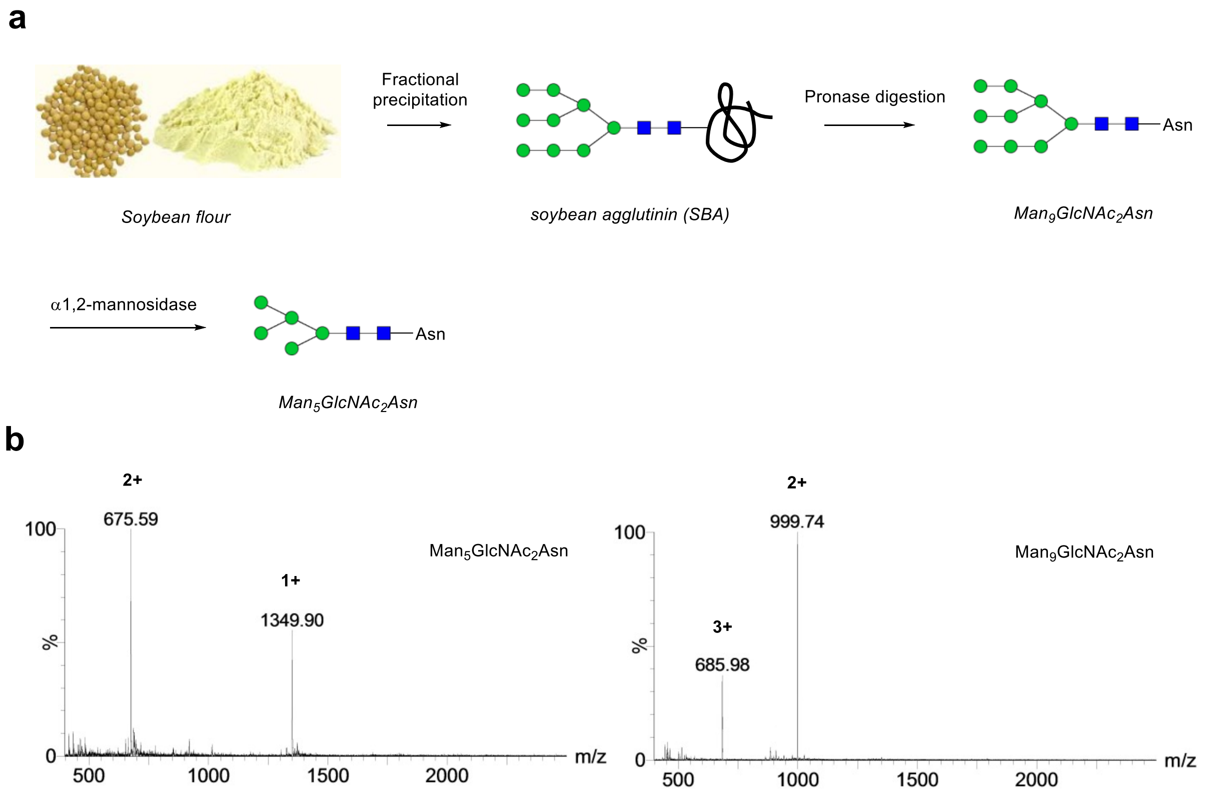

Supplementary Figure 7 | Biochemical Synthesis of $Man_5GlcNAc_2Asn$ and $Man_9GlcNAc_2Asn$. a Scheme showing the chemical synthesis pathway of $Man_5GlcNAc_2Asn$ and $Man_9GlcNAc_2Asn$. **b** ESI Mass Spectrum of purified $Man_5GlcNAc_2Asn$ and $Man_9GlcNAc_2Asn$.

SUPPLEMENTARY REFERENCES

1. van Aalten, D. M. F. *et al.* Structural insights into the catalytic mechanism of a family 18 exo-chitinase. *Proc. Natl. Acad. Sci.* **98**, 8979 L – 8984 (2001).
2. Williams, S. J., Mark, B. L., Vocadlo, D. J., James, M. N. G. & Withers, S. G. Aspartate 313 in the Streptomyces plicatus hexosaminidase plays a critical role in substrate-assisted catalysis by orienting the 2-acetamido group and stabilizing the transition state. *J. Biol. Chem.* **277**, 40055–40065 (2002).
3. Vaaje-Kolstad, G. *et al.* Interactions of a Family 18 Chitinase with the Designed Inhibitor HM508 and Its Degradation Product, Chitobiono-delta-lactone. *J. Biol. Chem.* **279**, 3612–3619 (2004).
4. Synstad, B. *et al.* Mutational and computational analysis of the role of conserved residues in the active site of a family 18 chitinase. *Eur. J. Biochem.* **271**, 253–262 (2004).
5. White, A. & Rose, D. R. Mechanism of catalysis by retaining β-glycosyl hydrolases. *Curr. Opin. Struct. Biol.* **7**, 645–651 (1997).
6. Jitonnom, J., Lee, V. S., Nimmanpipug, P., Rowlands, H. A. & Mulholland, A. J. Quantum mechanics/molecular mechanics modeling of substrate-assisted catalysis in family 18 chitinases: Conformational changes and the role of Asp142 in catalysis in ChiB. *Biochemistry* **50**, 4697–4711 (2011).
7. Santana, A. G., Vadlamani, G., Mark, B. L. & Withers, S. G. N -Acetyl glycals are tight-binding and environmentally insensitive inhibitors of hexosaminidases. *Chem. Commun.* **52**, 7943–7046 (2016).
8. Coines, J., Alfonso-Prieto, M., Biarnes, X., Planas, A. & Rovira, C. Oxazoline or Oxazolinium Ion? The Protonation State and Conformation of the Reaction Intermediate of Chitinase Enzymes Revisited. *Chemistry* **24**, 19265 (2018).
9. Liu, T. *et al.* The deduced role of a chitinase containing two nonsynergistic catalytic domains. *Acta Crystallogr. Sect. D Struct. Biol.* **74**, 30–40 (2018).
10. Aronson, N. N. *et al.* Family 18 chitinase-oligosaccharide substrate interaction: Subsite preference and anomer selectivity of Serratia marcescens chitinase A. *Biochem. J.* **376**, 87–95 (2003).
11. Papanikolau, Y. *et al.* High resolution structural analyses of mutant Chitinase A complexes with substrates provide new insight into the mechanism of catalysis. *Biochemistry* (2001). doi:10.1021/bi010505h
12. Vocadlo, D. J. & Davies, G. J. Mechanistic insights into glycosidase chemistry. *Current Opinion in Chemical Biology* (2008). doi:10.1016/j.cbpa.2008.05.010
13. Tews, I. *et al.* Bacterial chitobiase structure provides insight into catalytic mechanism and the basis of Tay-Sachs disease. *Nat. Struct. Biol.* **3**, 638–648 (1996).
14. Chen, L. *et al.* Structural characteristics of an insect group i chitinase, an enzyme indispensable to moulting. *Acta Crystallogr. Sect. D Biol. Crystallogr.* **70**, 932–942 (2014).
15. Malecki, P. H., Raczynska, J. E., Vorgias, C. E. & Rypniewski, W. Structure of a complete four-domain chitinase from Moritella marina, a marine psychrophilic bacterium. *Acta Crystallogr. Sect. D Biol. Crystallogr.* **69**, 821–829 (2013).
16. Ranok, A., Wongsantichon, J., Robinson, R. C. & Suginta, W. Structural and thermodynamic insights into chitooligosaccharide binding to human cartilage chitinase 3-like protein 2 (CHI3L2 or YKL-39). *J. Biol. Chem.* **290**, 2617–2629 (2015).
17. Hsieh, Y. C. *et al.* Crystal structures of Bacillus cereus NCTU2 chitinase complexes with chitooligomers reveal novel substrate binding for catalysis: A chitinase without chitin binding and insertion domains. *J. Biol. Chem.* **285**, 31603 (2010).

18. Klontz, E. H. *et al.* Molecular basis of broad spectrum N-glycan specificity and processing of therapeutic IgG monoclonal antibodies by Endoglycosidase S2. *ACS Cent. Sci.* **5**, 524–538 (2019).
19. Cremer, D. & Pople, J. A. A General Definition of Ring Puckering Coordinates. *J. Am. Chem. Soc.* **97**, 1354–1358 (1975).
