# Supplementary material for: Structural basis of mammalian high-mannose N-glycan processing by human gut Bacteroides
Source: Nat Commun. 2020 Feb 14;11:899. doi: 10.1038/s41467-020-14754-7 (PMC7021837; doi:10.1038/s41467-020-14754-7)
Supplement: Supplementary file 2 — Description of Additional Supplementary Files [file 41467_2020_14754_MOESM2_ESM.pdf]

Description of Additional Supplementary Files

File Name: Supplementary Data 1
Description: Identification of predicted PULs associated with the Bacteroidetes sequence homologues of EndoBT-3987
